# The Harvest-Time for Voluntary Hospitals

**Published:** 1918-09-21

**Authors:** 


### THE HARVEST-TIME FOR VOLUNTARY HOSPITALS.

In recent years a few of the larger hospitals in the Metropolis and some provincial centres of importance have adopted a suggestion, we have insisted on for years, to make special and most successful efforts to boom their hospital work, and extend the area of interest in that work through the quarterly courts of Governors. To secure this aim an able speaker has been invited to proclaim the work with eloquence and tact, and we have given full publicity in The Hospital to these efforts whenever made. The London Hospital, widely recognised for the publicity it secures for itself and its workers, has made this method of propaganda a living feature in its regular work, as our columns have demonstrated. Leicester, Cambridge, Norwich, to name only a few provincial hospitals, have found the practice which we have co-operated in making successful most valuable in results, so far as money and public interest are concerned. With October The Hospital commences a new volume, and the next two months, when a vigorous financial propaganda is instituted, always prove to be amongst the most fruitful in returns for hospitals by those who understand their business. We have, therefore, much pleasure in intimating our willingness to co-operate by giving publicity to those institutions of importance which may decide to make the experiment of booming their quarterly courts. This should be strengthened by a vigorous effort to extend the yield in contributions to, the interest of all classes in, and the waking up of the whole machinery and management of the hospital for the efficient maintenance of which those interested may be directly or indirectly responsible.

People whose work for the Empire, the Old Country, or locally lies in some portion of the hospital field can do no greater service to their institution, the community amongst which they reside, and themselves than to resolve to give thought, study, and continual work to the promotion of an active system of propaganda whereby all the money which every voluntary hospital may require can readily be secured from the people. We are confident, if they once begin actively to support the hospital in their midst, they will continue to support it after the war, and with ever-increasing interest thenceforward throughout their lives. This is the universal experience of hospital workers of the best, and we can endorse it with the full strength of fifty continuous years of money raising, which demonstrates that if the management of a hospital remembers every year those who have once sent a contribution, the latter will, as the years pass, come to welcome the secretary's communication. Indeed, the interest deepens until they grow to regard it as a great compliment that they are not forgotten by the administrators of the institution to which some circumstance or accident may have made them send their original contribution. Now is the time to work with stout faith and steady persistence. Remember the yeoman's work done at Cardiff. Set to work to make the yield of your own field, at least as fruitful. It means work and brains, of course. But, who that lacks them and the necessary energy, can voluntarily continue longer to hamper the progress of their voluntary hospital? The present times impose the duty on every man and woman to be up and always at their very best.

